# Incidence and nonunion rates of tibial fractures in adults with osteogenesis imperfecta: a retrospective cohort study of 402 patients with 42 fractures at an expert clinic

**DOI:** 10.1186/s12891-022-05966-7

**Published:** 2022-12-09

**Authors:** Simone Amber Munk, Gerrit Jan Harsevoort, Koert Gooijer, Mireille Angélique Edens, Antonius Adrianus Franken, Guus Johannes Maria Janus

**Affiliations:** 1 Department of Orthopedic Surgery, Isala, Zwolle, The Netherlands; 2 Epidemiology Unit, Department Innovation and Science, Isala, Zwolle, The Netherlands; 3 Department of Internal Medicine, Isala, Zwolle, The Netherlands

**Keywords:** Osteogenesis imperfecta, Tibial fracture, Nonunion, Adult

## Abstract

**Background:**

Tibial fractures are the most common fractures seen in adults and lead to the most nonunions. Osteogenesis imperfecta (OI) is characterized by increased bone fragility and higher risk of fractures. No studies have been published on the incidence of tibial fractures and nonunions in adults with OI. This study aims to summarize the incidence of tibial fractures and nonunions in this population.

**Methods:**

A retrospective, descriptive study. All medical charts of adult patients in the OI database of our OI expert clinic were analyzed for tibial fractures between 2008 and 2020. Tibial fracture incidence, nonunion rate, treatment modality and potential risk factors were determined.

**Results:**

The database consisted of 402 patients, 34 of whom had suffered one or more tibial fractures, resulting in 42 fractures. The incidence of tibial fractures in adults with OI is 870 per 100,000 person-years. Two out of 42 fractures led to nonunion (5%). It was not possible to adjust for risk factors or type of treatment.

**Conclusion:**

There is a higher incidence of tibial fractures in patients with OI, but a nonunion rate comparable to the general population. With only two nonunions it is not possible to draw conclusions on the influence of risk factors or treatment of tibial fractures on OI.

## Background

Osteogenesis imperfecta (OI), also known as brittle bone disease, is a genetic disorder most often caused by alterations in type I collagen that encompasses a heterogenous group of inherited bone dysplasias [1]. Among other abnormalities, it impacts quality of bone structure, resulting in an increased risk of long bone fractures and skeletal deformation [1]. Sillence made a classification based on clinical variety including types I-IV. In 2009 type V was added to the classification. The types have a variety in bone fragility, with type II being the worst and lethal prenatally, followed by type III showing skeletal deformity, and types V, IV, and I being the least at risk for fractures [2].

Treatment of fractures in OI is difficult due to the porous quality of the bone and a wide range of skeletal deformities [1, 3]. It can lead to complications and failure because of its challenging nature [4]. A study on femoral fractures in adults with OI concluded that up to 20% of these fractures led to nonunion after treatment [5], which increases the difficulty of fracture treatment for these patients.

In the general population, tibial fractures are the most common long bone fractures, also resulting in nonunion more often than other long bone fractures. Nonunion rates of tibial fractures vary through the ample literature; this may be partly due to different definitions of nonunion used [6–9]. To our knowledge, no research has been conducted on treatment of tibial fractures and nonunion rates in adults with OI.

Tibial fractures already have a high risk of developing nonunion. Knowing that femoral fractures often lead to nonunion in adults with OI [5], we expect tibial fractures in OI to lead to even more nonunions. This descriptive study follows Goudriaan et al. [5] in summarizing the incidence and nonunion at our expert clinic in Zwolle, the Netherlands, but now in tibial fractures in adults with OI. The study likewise aims to give advice on treatment of tibial fractures in OI.

## Method

### Study design and setting

We conducted a retrospective observational cohort study in an OI expert clinic for adults in Zwolle. Data covering January 2008 to February 2020 were retrieved.

### Study population

Medical data of all patients 18 years or older with OI were retrieved. This yielded 402 patients for 2008–2020. Inclusion criteria were diagnosis of OI and age 18 years or older at the time of the tibial fracture (Table 1). Exclusion criteria were use of corticosteroids, a second genetic syndrome, primary osseous tumor, or metastatic disease, as these could influence the risk of fracture and nonunion [7]. Death before finishing treatment was another exclusion criterion since union and adequacy of treatment could not be assessed (Table 1), wherein a finished treatment is defined as a healed fracture or proven nonunion (treated or untreated).

### Data and data collection

Data collection was aided by using a medical business intelligence tool (CT/cue). The records of all patients were searched with CT/cue by using the following keywords (in Dutch): lower leg fracture, tibial fracture, intramedullary pen tibia and intramedullary pen lower leg, plate fixation tibia, and plate fixation lower leg.

#### Data

As in Goudriaan et al. [5], data selected from the patient records included demographic characteristics and type of OI: both genetic and clinical type according to Sillence were included. Type of fracture according to the AO/OTA Fracture and Dislocation Classification [10] and treatment of the fracture (conservative and operative) including outcome (union, nonunion) and complications (infection, malposition, failure of osteosynthesis) were recorded. Potential risk factors for nonunion were identified, including bisphosphonate use, smoking, nutritional deficiency, vitamin D deficiency, mobilization status, metabolic disease, and endocrine pathology. Union was defined as the presence of bridging callus in at least three of four cortices, evaluated on two transverse levels on radiographic imaging. Nonunion was defined as non-radiographic changes to union or the absence of bridging callus of two or more cortices, evaluated on radiographs in two transverse levels for at least six months after treatment [5, 11].

In case of missing data, patients were asked for permission to retrieve the missing documentation from other hospitals. Information was only obtained when the patient’s consent to requesting data for research was documented. All participants of the study provided consent.

**Table 1:** Inclusion and exclusion criteria

Inclusion	Exclusion
Tibial fracture	No tibial fracture
Age ≥ 18 years	Age < 18 years
Osteogenesis imperfecta	Use of corticosteroids
	Death before full healing of fracture
	Second genetic syndrome, primary osseous tumor, or metastatic disease

### Statistical methods

This study contains a relatively small number of subjects with too little data to analyze statistically, therefore a descriptive study was conducted. Descriptive statistics were used to analyze the results using SPSS (Statistical Package for the Social Sciences). Categorical variables were expressed as percentage and metric variables as mean and standard deviation. Incidence rate was calculated over all 402 patients and the 12 years of inclusion (2008–2020). Incidence was calculated on number of fractures, not individuals.

### Ethics

The Medical Ethics Committee of Isala Hospital, Zwolle, The Netherlands, approved the study protocol and provided a non-WMO (Medical Research Involving Human Subjects Act) waiver (METC no. 200,638). All patients provided written consent for accessing their medical records, for obtaining additional records from other institutions, and for use of this information for research.

## Results

**Fig. 1:**
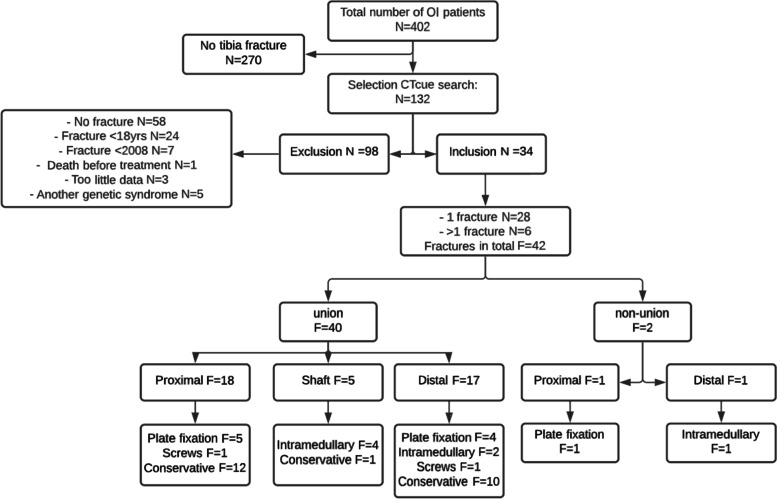
Flowchart of participants and fracture selection, union classification, and treatment of fractures. N = number of participants F = number of fractures

Of the 402 patients with OI in the database during the stated study period, 132 were selected with potential tibial fractures using CT/cue. The remaining 270 patients were assumed not to have had a tibial fracture since none of the search terms were found in their records. The dossiers of these 132 patients were reviewed in full by one researcher (SAM); 98 patients were excluded (Fig. 1) and 34 patients meeting the study criteria were included. Participants’ demographics are shown in Table 2.

Six participants had more than one tibial fracture over time and were included separately, resulting in a total of 42 fractures. Of these fractures, two resulted in nonunion after treatment.

**Table 2:** Demographic characteristics of all subjects with one or more tibial fractures

	All subjects N = 34
Age (years)	41 [32;61]
Gender (male)	17 (50)
Smoking	10 (29)
OI type	
I	22 (65)
III	6 (17)
IV	5 (15)
V	1 (3)

Data are presented as: median [quartile 1;quartile 3] or number of patients (percentage)

### Incidence of tibial fracture and nonunion

The 2008–2020 cohort of 402 OI patients included 42 fractures. This is an incidence of 870/100,000 tibial fractures annually. Two out of 42 fractures (5%) developed into nonunion. The annual incidence for nonunion is 38/100,000.

### Influence of OI type on healing rate

Twenty-six (62%) tibial fractures were seen in Sillence type I OI patients, 7 (17%) in type III, 8 (19%) in type IV, and one (2%) in type V OI patients. Of the 42 analyzed fractures, two resulted in nonunion (Table 3): an OI type I proximal fracture and an OI type III distal fracture.

### Influence of treatment on healing rate

**Fig. 2:**
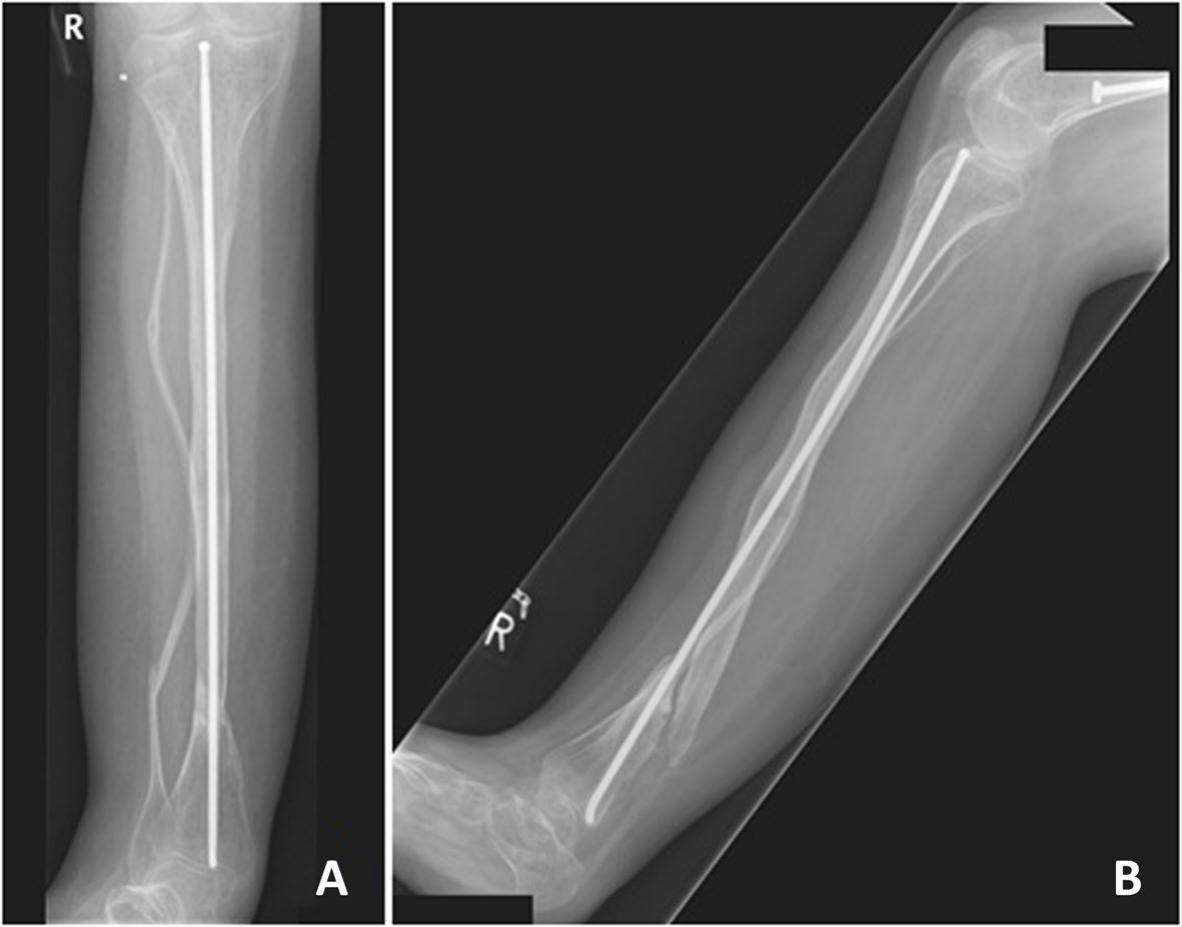
Nonunion distal tibial fracture, two years after initial treatment. **A** AP and **B** lateral view

For OI type I, only one out of 26 fractures (4%) resulted in nonunion and was initially treated with a plate fixation. The other nonunion was a type III OI distal tibial fracture – one out of seven fractures (14%). As treatment, a Rushpin was removed and a titanium elastic nail (TEN) was implanted. With no evidence of healing, additional cast immobilization was initiated with no result of bone healing. Since the patient is wheelchair-bound and had little pain, eventually the pseudarthrosis was accepted and the patient uses a brace for support (Fig. 2).

From another perspective, one out of five (20%) plate fixations for proximal tibial fractures led to nonunion. As for distal intramedullary nailing, one out of three (33%) led to nonunion. Conservative treatment alone (18 fractures) never led to nonunion, regardless of fracture location.

**Table 3:** Characteristics of patients with nonunions

	Gender	Age range at fracture	OI type	Fracture type	Primary treatment
1	Male	18–24	I	Proximal tibia	Plate fixation
2	Male	18–24	III	Distal tibia	Intramedullary fixation

## Discussion

In our study, the incidence of tibial fractures is 870/100.000 annually in adults with OI; we included 42 tibial fractures out of 402 patients (10%), with two nonunions (5%). To our knowledge, this is the first study to describe the incidence and number of nonunions in tibial fractures in adults with OI. We also aimed to draw conclusions as to whether OI treatment or type has any impact on the risk of nonunion, but with only two nonunions with different treatments this was not feasible.

Tibial fractures are the most common fractures in the general population. Hemmann et al. [12] recently estimated their incidence at 6–101/100,000 annually in the general population. In our OI population the incidence is 870/100,000 annually, thus higher than the general population. However, both their study and ours are most likely underestimating reality. They only included hospital patients, and in this study not all fractures of the OI population we studied might be known in our patient records: if treated elsewhere and not communicated with the expert clinic, the fracture would not have been traceable for this study. In addition, the exclusion criteria applied and the use of specific search terms in the selection of participants could have led to an underestimation of incidence.

Tibial fractures are also seen with most nonunions, even in the general population, ranging from 1 to 23% [7, 8, 13, 14]. Our findings of 5% show a comparable nonunion rate. The outcome differs from our expectation: we assumed the nonunion rate would be higher in OI. Important to note is that a tibial fracture is often the result of a high-energy trauma [7, 8, 14]. A study on tibial shaft fractures showed that the mechanism of injury and amount of soft tissue damage indeed enhanced the risk of nonunion [15]. In our study, the fractures were all the result of low-energy trauma, therefore the results are not directly comparable: if corrected for intensity of trauma, the nonunion rate might be lower than in the known literature on the general population.

### Strengths and limitations

This is a retrospective study where patient data was searched by using specific terms, therefore we cannot rule out the possibility of having missed fractures in the database. Besides, not all tibial fractures might be documented in our database, especially those treated successfully elsewhere. However, since Zwolle is the expert clinic on OI, it is to be expected that nonunions would have been referred to or at least discussed with our clinic and therefore would most likely be in our database.

As the fractures were treated in various hospitals and were of a different AO/OTA classification, various treatments were given. This gives a wider overview of risk of nonunion, yet further narrows the possibility of direct comparisons between treatment and thus advice on how to treat these fractures.

By stating a clear definition of nonunion that can be objectively measured on X-ray, there is no question on whether the fracture healed. With the consent of all patients it was possible to retrieve most missing data required to determine union. All the included fractures had reached union or were stated as nonunion at least one year after starting treatment.

### Patient-dependent factors

Given the retrospective nature of this study, we were able to include risk factors: bisphosphonate use, smoking, nutritional and vitamin D deficiency, mobilization status, and other metabolic diseases. Unfortunately, data were incomplete and despite efforts made not all data could be retrieved, as it was often undocumented. In addition, there were only two nonunions in the current study, making it impossible to adjust for these risk factors, even if the data had been complete.

## Conclusion

This study set out the incidence and nonunion rate of tibial fractures in the population of adults with OI known at our expert clinic. Whereas the incidence of tibial fractures is higher in the OI population, the nonunion rate is comparable to the general population, although there might be a difference if type of injury and soft tissue damage were taken into account. With only two nonunions, we could draw no hard conclusions as to whether type of OI, type of treatment, or risk factors influence the risk of nonunion in tibial fractures. Further research, preferably multicenter, must be conducted to make an adequate statement. More data may facilitate giving useful advice on how to treat a nonunion, leading to better care for the OI population.

## Data Availability

The data used and analyzed for the current study are available from the corresponding author upon reasonable request.

## Abbreviations

OI: Osteogenesis imperfecta
CT/cue: Medical business intelligence tool - a search engine for data in patient charts
AO/OTA: Association of Osteosynthesis Foundation/Orthopaedic Trauma Association
SPSS: Statistical Package of the Social Sciences
Non-WMO waiver: A waiver that states that the study is not subject to the Medical Research involving Human Subjects Act (WMO)
METC: Medical Ethics Committee
TEN: Titanium Elastic Nail
